# Comprehensive Evaluation of Quality of Life in Penile Cancer Patients following Surgical Treatment

**DOI:** 10.3390/healthcare11233091

**Published:** 2023-12-04

**Authors:** Darko Jovanović, Miodrag Aćimović, Tomislav Pejčić, Bogomir Milojević, Bojan Čegar, Milica Zeković, Nikola Lisičić, Zoran Džamić, Gorica Marić

**Affiliations:** 1 Clinic for Urology, University Clinical Center of Serbia, 11000 Belgrade, Serbiamiodrag.acimovic@med.bg.ac.rs (M.A.);; 2 Faculty of Medicine, University of Belgrade, 11000 Belgrade, Serbia; 3 Centre of Research Excellence in Nutrition and Metabolism, Institute for Medical Research, National Institute of Republic of Serbia, University of Belgrade, 11000 Belgrade, Serbia; zekovicmilica@gmail.com

**Keywords:** erectile dysfunction, EORTC QLQ-C30, urological malignancy, hospital anxiety and depression scale

## Abstract

Background: Penile cancer (PC) is a highly aggressive disease, with a significant tendency for lymphatic spreading and subsequent development of distant metastases. The mutilating nature of PC surgical treatment has profound implications on the patient’s body integrity and self-image, sexual life and intimacy, voiding and mental health. The aim of our study was to comprehensively evaluate PC patients’ post-treatment quality of life (QoL), sexual activity, self-esteem, fatigue and fear of disease recurrence. (2) Methods: A cross-sectional study was conducted at the Clinic of Urology, University Clinical Centre of Serbia, and included 31 PC patients. Data were collected by means of a questionnaire. (3) Results: The average score on the Global health status scale was 67.2 out of 100 (ranging from 16.7 to 100), and the SD was 22.5. Hierarchical linear regression analysis showed that demographic characteristics, Hospital Anxiety and Depression scale (HADS) anxiety and depression scores, total Multidimensional Fatigue Inventory, Fear of cancer recurrence and Rosenberg scores and erectile function score explained a total of 78.2% of the variance in the global health status/QoL scale of PC patients. (4) Conclusions: Efforts should be made not only to increase the survival of PC patients after surgical treatment but also to enable the best possible level of QoL in the post-operative period.

## 1. Introduction

Penile cancer (PC) is a rare urological malignancy. In Western industrialized countries, it is an uncommon diagnosis, with an overall incidence of 1 per 100,000 men per year. In contrast, this cancer has a substantially greater incidence in developing South American, African and Southeast Asian countries, thus being an important public health problem [1]. Although PC is often diagnosed in men in their sixth decade, it can also affect younger populations. It is estimated that 20% of PC patients are under the age of 40, and 7% are under the age of 30 [2]. One of the major risk factors for PC development is human papillomavirus (HPV) infection, which is associated with 30% to 40% of invasive PCs [3]. Other recognized risk factors include phimosis and chronic penile inflammation, smoking, low socioeconomic status, multiple sexual partners and ultraviolet phototherapy [4]. 

PC is a highly aggressive disease, with a significant tendency for lymphatic spreading in regional lymph nodes and subsequent development of distant metastases. While 5-year cancer-specific survival probability (CSS) is 85%, the local recurrence rate ranges between 4% and 18% [5]. Accordingly, PC treatment must be conducted in a timely manner and with the utmost adherence to accepted surgical oncological principles. Traditional surgical management of PC consisted of partial or total penectomy. As an alternative to penectomy, organ-preserving surgical techniques have been recently developed, providing comparable oncological outcomes [6]. Nevertheless, the mutilating nature of PC surgical treatment has profound implications on the patient’s body integrity and self-image, sexual life and intimacy, voiding and mental health [7]. 

Although PC therapy has a considerable impact on patients’ sexual activity, self-esteem and quality of life (QoL), the rarity of this cancer contributes to the lack of published literature on this topic. Additionally, there are no standardized tools and metrics for the assessment of these patients. The majority of the studies indicate compromised post-treatment sexual and urinary function results and QoL, while the severity of the symptoms is highly correlated with the extent of the primary tumor surgery [8]. 

Based on the aforementioned, and keeping in mind that this kind of research has not been performed before in the Serbian population of PC patients, the aim of our study was to comprehensively evaluate post-treatment QoL, sexual activity, self-esteem, fatigue and fear of disease recurrence in PC patients in Belgrade, Serbia.

## 2. Materials and Methods

### 2.1. Study Design and Participants

This investigation employed a cross-sectional research design and was carried out at the Clinic of Urology, University Clinical Centre of Serbia, situated in Belgrade, the capital city of Serbia. As the largest healthcare facility in the country, it plays a vital role in providing specialized medical services for the local population of the city of Belgrade, while serving as the pivotal referral hub for the entire country. 

From 1 January 2022 to 15 June 2023, a total of 31 eligible PC patients were included in the study. Eligible individuals were those with a confirmed diagnosis of PC based on histopathological examination and clinical evaluation and aged 18 years or older. Exclusion criteria were applied to individuals who met any of the following conditions: a history of malignancies other than PC; significant cognitive or neurological impairment that could hinder their ability to independently complete questionnaires accurately; mental health conditions or substantial functional limitations that might compromise the validity of the study’s results; current psychiatric disorders necessitating ongoing treatment; language barriers or limited proficiency in effectively communicating in Serbian, which served as the primary language for data collection; and participants with terminal conditions or limited life expectancy unrelated to PC. Exclusion criterion was also patients’ refusal to participate in the study. To minimize the burden on participants and maximize recruitment rates, data collection was coordinated during the participants’ routine outpatient visits, thereby eliminating the need for extra clinic appointments. 

### 2.2. Ethical Considerations

The study was conducted with adherence to international ethical standards for research involving human subjects outlined in the Declaration of Helsinki and was formally approved by the Ethics Committee of the University Clinical Centre of Serbia (No. 250/3, 25 May 2023). Written informed consent was obtained from all participants prior to their enrollment in the study.

### 2.3. Data Collection

Demographic characteristics of patients were collected by means of a short questionnaire made for the purposes of the study and included their age, marital status, data on children, place of residence, living arrangement, years of education and working status. Data on clinical characteristics were extracted from the patients’ medical records and comprised age at diagnosis, disease duration, time from symptom onset to diagnosis and time between diagnosis and surgical treatment. 

In accordance with the objectives, this study employed a comprehensive approach to assess various aspects of quality of life and psychological well-being in PC patients, encompassing multiple validated questionnaires and evaluations.

The European Organization for Research and Treatment of Cancer (EORTC) QLQ-C30 is a 30-item questionnaire designed for the measurement of health-related quality of life in cancer patients [9]. Single items produce five functional scales, three symptom scales, a global health status/QoL scale and six items which are scored separately. The first step in scoring scales is calculating raw scores. A raw score is obtained by summing corresponding items and dividing by the number of items in a certain scale. Then, each scale score is linearly transformed to a 0–100 scale with higher scores indicating better quality of life (a global health status/QoL scale), better functioning (functional scales) or higher level of symptoms (six single items related to symptoms).

The international index of erectile function (IIEF) was used to detect the presence and categorize the severity of erectile function [10]. It contains 15 items covering the following dimensions: erectile function (6 items), orgasmic function (2 items), sexual desire (2 items), intercourse satisfaction (3 items) and overall sexual satisfaction (2 items). Based on the erectile function (EF) domain score (ranging from 1 to 30), erectile dysfunction (ED) is classified into the following five categories: no ED (EF score 26 to 30), mild (EF score 22 to 25), mild to moderate (EF score 17 to 21), moderate (EF score 11 to 16) and severe (EF score 1 to 10).

Rosenberg self-esteem scale is a short, self-report, 10-item questionnaire, developed in 1965 [11]. Items are scored using a 4-point Likert scale (from “Strongly agree” to “Strongly disagree”). A total score is obtained by summing all items. A higher total score denotes a higher level of self-esteem.

In order to assess the presence and level of fatigue in PC patients, the Multidimensional Fatigue Inventory (MFI) was used [12]. It is a self-reported tool containing 20 items on a five-point Likert scale and divided into five domains: General Fatigue, Physical Fatigue, Mental Fatigue, Reduced Motivation and Reduced Activity. Scale scores range between 4 and 20, and total fatigue scores can have values between 20 and 100. Higher scores mean a higher level of fatigue. 

Hospital Anxiety and Depression scale (HADS) [13] was used to assess anxiety and depression symptoms in PC patients. The scale has 14 items (seven assessing anxiety symptoms and seven assessing depression symptoms) ranging from 0 to 3. Scores are separately calculated for anxiety and depression (ranging from 0 to 21 for each scale). Total scores are interpreted as normal (0–7), mild (8–10), moderate (11–15) and severe (16–21). 

Finally, the fear of cancer recurrence among PC patients was determined by the self-report Fear of Cancer Recurrence Inventory (FCRI) [14]. A total of 42 items correspond to seven different domains: triggers (8 items), severity (9 items), psychological distress (4 items), coping strategies (9 items), functioning impairments (6 items), insight (3 items) and reassurance (3 items). Summing all the scales leads to the FCR total score where increasing values represent a greater fear of cancer recurrence.

All data were initially analyzed using descriptive statistics (mean and standard deviation for continuous variables, median and interquartile range for ordinal variables and frequencies and percentages for nominal data). Correlations between variables were determined using Pearson and Spearman correlation coefficients. In order to assess the main contributors to health-related QoL in PC patients, hierarchical linear regression analysis was performed. In this analysis, the global health status/QoL scale was set as the dependent variable. Independent variables in the first model were demographic characteristics, age, marital status (married vs. other) and number of children. In the second model were added HADS anxiety and depression scores and total MFI, FCR and Rosenberg scores. Finally, the variable added in the third model was erectile dysfunction.

*p* values less than 0.05 were considered significant. All analyses were performed using Statistical Package for Social Sciences (SPSS), version 20.0.

## 3. Results

### 3.1. Sample Characteristics

The demographic and clinical characteristics of PC patients are reported in Table 1. The average age of PC patients was 66.9 ± 9.1 years (range 44–89). The majority of them were married (74.2%), with a median of 12 years of education, living with their family (64.5%) in an urban area (74.2%) and retired (61.3%) (Table 1). 

In terms of clinical characteristics, PC patients in our cohort on average had 64.2 ± 9.0 years at diagnosis, and the mean disease duration was 2.8 ± 3.0 years (Table 1). The average time to diagnosis was 9.2 ± 17.4 months, and the average time from PC diagnosis to surgical treatment was 4.3 ± 6.2 months. Data on the type of treatment were available for 24 PC patients. Among them, six (19.4%) underwent total penectomy, ten (32.3%) partial penectomy and five (16.1%) glansectomy. One patient (3.2%) received topical treatment, and two (6.5%) had excision with resurfacing (data not presented). Eighteen patients (58.1%) had some comorbid condition (Table 1).

### 3.2. Quality of Life

The average score on the Global health status scale was 67.2 out of 100 (ranging from 16.7 to 100), and the SD was 22.5 (Table 2). Among functional scales, the highest score was obtained for scales Cognitive functioning (92.5 ± 14.8) and Emotional functioning (89.2 ± 18.5). The lowest score was shown for the Physical functioning scale (84.9 ± 25.4) (Table 2). For symptom scales, the highest score (indicating highest symptom burden) was obtained for Fatigue (16.1 ± 25.1), and the lowest score (indicating lowest symptom burden) was found in the case of Nausea and vomiting (2.2 ± 7.1) (Table 2).

### 3.3. Anxiety and Depression 

Mean HADS anxiety and depression total scale scores were 4.9 ± 2.8 (range 0–14) and 4.1 ± 3.6 (range 0–10), respectively. Four PC patients (12.9%) were classified as having mild anxiety symptoms (score between 8 and 10), one patient (3.2%) had moderate anxiety, and the remaining twenty-six (83.9%) scored within the normal range (≤8). Mild depressive symptoms (score between 8 and 10) were found in nine PC patients (29.0%), and the remaining twenty-two (71.0%) PC patients scored less than 8 (normal range) (data not presented). 

### 3.4. Fatigue

Mean MFI total scores and subscale scores are shown in Table 3. The average total MFI score was 43.6 ± 17.0 (range 20–91). The highest level of fatigue was recorded for the Physical fatigue scale (10.1 ± 4.3) and General fatigue (9.5 ± 4.1). The lowest level of fatigue was observed for the Mental fatigue scale (7.1 ± 3.3) (Table 3). 

### 3.5. Self-Esteem

None of the PC patients had a low level of self-esteem (total score < 15), as indicated by the Rosenberg self-esteem scale score. Scores ranged from 15 to 29, and the average score was 24.2 ± 3.2. 

### 3.6. Presence of Erectile Dysfunction

The presence of severe erectile dysfunction was registered in the majority of PC patients (19/31, 61.3%), and only six PC patients (19.4%) were without symptoms of erectile dysfunction (Table 4). 

### 3.7. Fear of Cancer Recurrence

Fear of cancer recurrence inventory answers yielded an average total score of 42.7 ± 34.6 (range 0–110). When scale scores were analyzed separately, the highest scores were observed for scales Coping strategies (11.0 ± 10.0) and Severity (9.2 ± 7.6) (Table 5). The lowest scores were obtained for the following scales: Insight (2.1 ± 3.0), Reassurance (2.8 ± 2.9) and Psychological distress (3.0 ± 3.4) (Table 5). 

Correlations between different demographic and clinical parameters of PC patients are demonstrated in Table 6. Older age of participants statistically significantly correlated with higher HADS anxiety (r = 0.415, *p* = 0.020) and depression scores (r = 0.394, *p* = 0.031), more severe erectile dysfunction (r = −0.507, *p* = 0.005) and lower Global health status scores (r = −0.399, *p* = 0.026) (Table 6). Place of residence (living in a rural area) strongly correlated with a higher FCR total score (r = 0.437, *p* = 0.014). Higher Global health status scores significantly correlated with lower total MFI scores (r = −0.603, *p* < 0.001), lower HADS depression scale scores (r = −0.779, *p* < 0.001) and lower HADS Anxiety scale scores (r = −0.362, *p* = 0.045). Additionally, a higher HADS Anxiety scale score correlated with a higher MFI total score (r = 0.439, *p* = 0.013), higher HADS Depression score (r = 0.500, *p* = 0.005) and higher total FCR score (r = 0.484, *p* = 0.006). Moreover, a higher HADS Depression scale score was associated with a higher total MFI score (r = 0.665, *p* < 0.001). A higher total MFI score was associated with a lower Rosenberg self-esteem scale score (r = −0.453, *p* = 0.012). Finally, a higher level of self-esteem correlated with a lower FCR total score (r = −0.615, *p* < 0.001). 

### 3.8. Hierarchical Regression Analysis

The results of hierarchical linear regression analysis are shown in Table 7. The hierarchical regression analysis showed that the demographic characteristics of PC patients (age, marital status (married vs. other) and number of children) accounted for 21.5% of the variance in the global health status/QoL scale score (*p* = 0.115) (Model 1). In the second model, when HADS anxiety and HADS depression scores, as well as total MFI, FCR and Rosenberg scores, were added, another 45.8% in the variance of the global health status/QoL scale score was explained (*p* = 0.002) (Table 7). Finally, when erectile dysfunction was included in Model 3, the proportion of variance explained increased by an additional 10.9% (*p* < 0.001). This means that the final model of hierarchical linear regression analysis showed that demographic characteristics (age, marital status (married vs. other) and number of children), HADS anxiety and depression scores, total MFI, FCR and Rosenberg scores and erectile function score explained a total of 78.2% of the variance in the global health status/QoL scale of PC patients in Belgrade, Serbia (Table 7).

## 4. Discussion

The main finding of our study is that the most significant contributors to health-related QoL among PC patients in Belgrade, Serbia, are age (*p* = 0.008) and the level of erectile dysfunction (0.008). In addition, the level of fatigue (*p* = 0.031), anxiety (0.044) and depression (0.030), as well as the level of fear of cancer recurrence (0.030), should be taken into consideration as important factors since they also reached statistical significance. It should be kept in mind that due to the small sample size (n = 31), some of these variables and also those that failed to reach it could have achieved higher statistical significance in a greater sample. 

The patient’s sexual life, including sexual desire, ability to obtain an erection and orgasm, frequency of intercourse and relationship with their partner are all significantly impacted by the surgical treatment of PC. Erectile dysfunction is a common urological condition that affects a significant number of men, with a prevalence that increases with age. It is defined as the consistent inability to achieve or maintain an erection sufficient for satisfactory sexual performance. Beyond the physical implications, erectile dysfunction can have a profound impact on the quality of life of affected individuals. In a systematic review that included 128 patients from six studies, Maddineni et al. reported a post-treatment decline in sexual function in two-thirds of PC patients [15]. Sansalone et al. demonstrated statistically significant differences between preoperative and post-operative IIEF scores for all categories of sexual function [7]. In the study conducted by D’Ancona and colleagues, reduced sexual function was observed in 36% of patients, while sexual desire, satisfaction and frequency of intercourse remained unchanged in the majority of patients [16]. Romero et al. detected erectile dysfunction in 44.4% of patients who underwent partial penectomy, while only 33.3% of patients retained pre-treatment sexual intercourse frequency and expressed satisfaction with overall sexual life [17]. Due to the highly mutilating nature of the procedure, sexual activity after total penectomy was observed in only 10% to 20% of patients [18]. Contrary, laser treatment of localized PC was associated with satisfying post-operative cosmetic results and preserved erectile function in the majority of patients [19]. In comparison to the published literature, our cohort of patients exhibited a high erectile dysfunction rate (80.6%), with a substantial percentage of patients having severe erectile dysfunction (61.3%) and only 19.4% of patients maintaining satisfactory erectile function. These findings could be attributed to the comparatively large proportion of patients with an advanced stage of disease, a subsequent significant number of total and partial penectomies and a low percentage of penile-preserving procedures.

Depression and other mental health issues have already been established as important determinants of PC patients’ QoL. Studies showed that half of PC patients experience some psychiatric symptomatology [15]. Similarly, it has been shown that depression can be present in 50% of PC patients [15]. The association between depression and the risk of suicide is well known. PC, on average, occurs in males in their sixth decade, and it has been reported that in elderly people, suicide most frequently occurs due to some health disorder, predominantly malignant disease [20]. Despite this fact, a study from the US revealed that although the risk of suicide is higher in PC patients compared to controls of the same age, this risk is not higher compared to patients with other urological malignant diseases as well as non-urological malignancies [21]. What is also surprising is that PC patients with a low grade and low stage of disease showed a higher risk of suicide compared to those PC patients with more advanced disease [21]. According to the same study, the risk of suicide was highest among males aged 50-59 years (SMR = 2.56, 95% CI 2.04–3.19) and those who were married (SMR = 2.05, 95% CI 1.57–2.64) (compared to those who were single, divorced or widowed) [21]. 

Changes in the QoL level of PC patients are driven to a significant extent by PC treatment modalities, and the burden of psychiatric symptoms increases with a more aggressive treatment approach [21]. This leads to alterations in sexual functioning as well as changes in patients’ body image and perception of their sexuality [22,23,24,25]. In a study conducted by Opjordsmoen et al., some PC patients reported that if it was possible, they would rather choose to live shorter after treatment but with more preserved sexual function [26]. In that light, organ-preserving surgical treatment in PC patients has been shown to be associated with better health-related QoL [27]. 

None of the PC patients in our study had low self-esteem. In deliberating the unforeseen absence of low self-esteem among the studied cohort of PC patients, several plausible explanations merit thorough exploration. Foremost, the resilience within this patient population implies their adaptive coping mechanisms in confronting the unique challenges associated with PC. Furthermore, the robust psychosocial support networks prevalent among study participants, whether from familial, social or support group avenues, could contribute significantly to fostering a positive self-esteem milieu. Cultural and societal factors may also wield influence, shaping distinct perceptions of self-esteem. Additionally, the possibility of patient selection bias cannot be discounted, as recruitment processes or inclusion criteria may unintentionally favor individuals with higher self-esteem. Moreover, the temporal aspect of the study in relation to patients’ cancer trajectories should be considered, recognizing the potential for adaptive processes over time. Only four PC patients (12.9%) had mild anxiety symptoms, and one (3.2%) had a moderate level of anxious symptoms. Nine PC patients (29.0%) had mild depression symptoms. This is of particular interest since ten PC patients (32.3%) had partial and six PC patients (19.4%) had total penectomy. One of the potential explanations for these findings could be cultural issues. Namely, it has been previously reported that readiness to speak about physical and mental health status may depend on norms and customs set in certain cultures [28]. In some cultures, it is common to speak about health issues while in others, it is considered a weakness to say that a person does not feel good, is tired or feels anxious or depressed. This is especially emphasized in the male population, and keeping in mind that PC is a male health issue, these circumstances should be taken into consideration when analyzing the results of this study. This can be even more highlighted in PC patients given that many persons do not feel comfortable speaking about their sexual health. Data on the presence of anxiety and depression among PC patients vary in different studies. D’Ancona et al. showed that among 14 PC patients who underwent partial penectomy, anxiety and depression were not a significant issue [16]. Contrary to our results, Ficarra et al. found that PC patients more frequently felt anxious than depressed [29]. 

The significance of our investigation is accentuated by acknowledging the pivotal role of the psychosocial dimensions in cancer, constituting an indispensable facet of patient care. A nuanced comprehension of the distinctive challenges confronted by survivors of PC is imperative to customize support mechanisms and interventions that cater to their particular requirements. The implications of our research extend beyond the realms of academic discourse, resonating with tangible enhancements in patient-centered care and the development of targeted survivorship programs tailored to the unique needs of individuals recovering from this urooncopathology.

The conclusions derived from our investigation should be considered with caution due to several limitations. Firstly, the study employed a cross-sectional design, precluding any inference of causality between the variables. Additionally, the relatively small sample size may restrict the generalizability of the findings. It is plausible that a larger sample would reveal additional correlations or trends beyond the scope of this research. Nevertheless, it is noteworthy to acknowledge that the modest sample size was largely attributable to the rarity of PC and the high mortality rate associated with the disease, which presents considerable challenges for recruiting and retaining participants in observational studies. The rationale behind the relatively short duration of the recruitment period stems from logistical considerations, practical constraints and the need to ensure the homogeneity of data collection within the defined timeframe. Moreover, self-reported data may introduce bias since they rely on personal perceptions and recollections, which are vulnerable to memory lapses, interpretation disparities and inclinations toward providing socially desirable responses rather than factual ones. Beyond the delineated limitations, it is imperative to recognize that the intricacies of the national context may introduce distinct variables influencing the study outcomes. Consequently, extrapolation to broader international contexts should be approached with caution.

Our research has several key strengths, including its comprehensive approach to assessing various aspects of QoL and psychological well-being in PC patients. The study also benefits from a robust methodological and statistical analytical framework designed to understand the intricate relationships between factors affecting these variables within such a distinct patient population. Notably, the study’s clinical relevance and real-world implications are significant, as it provides valuable insights for clinicians and healthcare providers to enhance survivorship care. Our findings emphasize the necessity for sustained psychosocial support for PC patients and highlight the importance of early detection and treatment of psychological distress. Conducted in Belgrade, Serbia, the study addresses a specific geographic region’s healthcare needs and underscores the importance of customizing patient care to address the unique challenges confronting PC patients in this setting. Finally, as PC is an orphan disease and an understudied area in oncology research, this study contributes valuable insights to the existing literature, offering a deeper understanding of the post-operative experiences of PC patients and adding to the overall knowledge base in urological oncology.

## 5. Conclusions

PC, although a rare malignancy, has the potential to interfere with many components of human well-being including QoL, social interactions and sexual functioning. These issues result from the disease itself but also from surgical treatment. Our results indicated that age and the level of erectile dysfunction play a key role in the QoL of PC patients and that the level of fatigue, depression, anxiety and fear of cancer recurrence should also be considered as a priority in PC patients’ management. These results should represent valid input for healthcare workers and policymakers. PC patients should have psychosocial support during all stages of their treatment. Along with treating their physical health, attention should be equally directed to preserving mental health through counseling and regular check-ups. Efforts should be made not only to increase the survival of PC patients after surgical treatment but also to enable the best possible level of QoL in the post-operative period. 

## Figures and Tables

**Table 1:** Demographic and clinical characteristics of the patients.

Variable	Observed Values
Age (years), X ± SD	66.9 ± 9.1
Marital status, n (%) Married Single Divorced Widowed	23 (74.2) 2 (6.5) 3 (9.7) 3 (9.7)
Number of children, n (%) None One Two or more	6 (19.4) 7 (22.6) 18 (58.0)
Living arrangement, n (%) Alone With partner With family	7 (22.6) 4 (12.9) 20 (64.5)
Place of residence, n (%) Urban Rural	23 (74.2) 8 (25.8)
Years of education (median, interquartile range)	12, 3.00
Working status Employed Unemployed Retired	11 (35.5) 1(3.2) 19 (61.3)
Age at diagnosis (years), X ± SD	64.2 ± 9.0
Disease duration (years), X ± SD	2.8 ± 3.0
Time to diagnosis (months), X ± SD	9.2 ± 17.4
Time from diagnosis to surgical treatment (months), X ± SD	4.3 ± 6.2
Comorbidities, n (%) Diabetes mellitus Hypertension Stroke Myocardial infarction Heart failure Chronic obstructive pulmonary disease	9 (29.0) 17 (54.8) 2 (6.5) 1 (3.2) 2 (6.5) 2 (6.5)

SD—standard deviation.

**Table 2:** Health-related quality of life of PC patients.

Scale	Items	Mean ± SD	Range
*Global health status*			
Global health status/QoL	29, 30	67.2 ± 22.5	16.7–100
*Functional scales*			
Physical functioning	1 to 5	84.9 ± 25.4	0.0–100
Role functioning	6, 7	85.5 ± 23.1	0.0–100
Emotional functioning	21 to 24	89.2 ± 18.5	33.3–100
Cognitive functioning	20, 25	92.5 ± 14.8	33.3–100
Social functioning	26, 27	85.5 ± 24.2	0.0–100
*Symptom scales/items*			
Fatigue	10, 12, 18	16.1 ± 25.1	0.0–100
Nausea and vomiting	14, 15	2.2 ± 7.1	0.0–33.3
Pain	9, 19	9.1 ± 14.2	0.0–50.0
Dyspnoea	8	7.5 ± 14.2	0.0–33.3
Insomnia	11	10.8 ± 21.8	0.0–100
Appetite loss	13	2.2 ± 12.0	0.0–66.7
Constipation	16	5.4 ± 19.4	0.0–100
Diarrhea	17	6.5 ± 20.0	0.0–100
Financial difficulties	28	15.1 ± 33.2	0.0–100

**Table 3:** Multidimensional Fatigue Inventory scale scores.

Scale	Mean Scale Score ± SD	Range
General fatigue	9.5 ± 4.1	4–20
Physical fatigue	10.1 ± 4.3	4–20
Reduced activity	8.9 ± 4.3	4–20
Reduced motivation	8.0 ± 3.4	4–15
Mental fatigue	7.1 ± 3.3	4–17
Total MFI score	43.6 ± 17.0	20–91

**Table 4:** Mean international index of erectile function scores.

Category	N (%)
Severe erectile dysfunction (score 1 to 10)	19 (61.3)
Moderate erectile dysfunction (score 11 to 16)	1 (3.2)
Mild to moderate erectile dysfunction (score 17 to 21)	2 (6.5)
Mild erectile dysfunction (score 22 to 25)	1 (3.2)
No erectile dysfunction (score 26 to 30)	6 (19.4)

**Table 5:** Fear of cancer recurrence inventory scale scores.

Scale	Score	Score Range
Triggers	7.6 ± 6.6	0–20
Severity	9.2 ± 7.6	0–30
Psychological distress	3.0 ± 3.4	0–10
Coping strategies	11.0 ± 10.0	0–29
Functioning impairments	7.0 ± 8.2	0–31
Insight	2.1 ± 3.0	0–11
Reassurance	2.8 ± 2.9	0–10
Total score	42.7 ± 34.6	0–110

**Table 6:** Correlation of different demographic and clinical characteristics of PC patients.

	MFI Total Score	HADS Anxiety	HADS Depression	Erectile Dysfunction	Rosenberg Total Score	Global Health Status	FCR Total Score
Age	r = 0.317	r = 0.415 *	r = 0.394 *	r = −0.507 **	r = 0.027	r = −0.399 *	r = −0.093
Place of residence	r = −0.117	r = 0.216	r = −0.130	r = −0.115	r = 0.015	r = 0.069	r = 0.509 **
HADS Anxiety	r = 0.439 *	/					
HADS Depression	r = 0.665 **	r = 0.500 **	/				
Erectile dysfunction	r = −0.359	r = −0.132	r = −0.260	/			
Rosenberg total score	r = −0.453 *	r = −0.190	r = −0.343	r = 0.075	/		
Global health status	r = −0.603 **	r = −0.362 *	r = −0.779 **	r = 0.089	r = 0.216	/	
FCR total score	r = 0.205	r = 0.484 **	r = 0.299	r = −0.104	r = −0.615 **	r = −0.220	/

Bold values denote statistical significance (* *p*-values < 0.05, ** *p*-values < 0.01).

**Table 7:** Results of hierarchical linear regression analysis.

Variable	Model 1			Model 2			Model 3		
	**B**	**SE (B)**	**β**	**B**	**SE (B)**	**β**	**B**	**SE (B)**	**Β**
Age	−0.84	0.51	−0.33	−0.59	0.45	−0.23	−1.38	0.46	−0.55 **
Marital status (married vs. other)	13.40	10.80	0.26	−1.49	8.95	−0.03	9.31	8.33	0.18
Number of children	−3.95	5.12	−0.15	−3.68	4.09	−0.14	−5.45	3.48	−0.21
HADS Anxiety				2.11	1.87	0.25	3.54	1.64	0.41 *
HADS Depression				−4.41	1.37	−0.71 *	−2.94	1.25	−0.47 *
MFI total score				−0.20	0.28	-0.15	−0.65	0.28	−0.48 *
Rosenberg total score				−1.54	1.45	−0.22	−2.54	1.26	−0.37
FCR total score				−0.24	0.18	−0.33	−0.38	0.16	−0.53 *
Erectile function							−1.03	0.34	−0.52 **
*R^2^*	0.22			0.67			0.78		
*F* for change in *R^2^*	2.190			4.881 **			7.172 **		

B—unstandardized coefficient; SE—standard error; β—standardized coefficient; bold values denote statistical significance (* *p*-values < 0.05, ** *p*-values < 0.01).

## Data Availability

Data supporting reported results can be obtained from the corresponding author on reasonable request.
